# Supplementary material for: The effects of fresh embryo transfer and frozen-thawed embryo transfer on the perinatal outcomes of single fetuses from mothers with PCOS
Source: PLoS One. 2024 Oct 28;19(10):e0312003. doi: 10.1371/journal.pone.0312003 (PMC11515985; doi:10.1371/journal.pone.0312003)
Supplement: Attachment — Submitted filename: 20240826-review comments reply letter.docx [file pone.0312003.s003.docx]

Dear Reviewers：

Thank you for your letter and for the reviewers' comments concerning our manuscript entitled “The effect of fresh embryo transfer and frozen-thawed embryo transfer on the perinatal outcomes of single fetuses from mothers with PCOS” (ID: PONE-D-24-09843R2）. Those comments are all valuable and very helpful for revising and improving our paper, as well as the important guiding significance to our researches. We have studied comments carefully and have made correction which we hope meet with approval. Revised portion are marked in yellow in the paper. The main corrections in the paper and the responds to the reviewer’s comments are as flowing：

Responds to the reviewer's comments:

**Reviewer#2:**

1. Gravity and Parity are still missing - these are certainly factors that could influence the conclusions of this paper. It is concerning that the authors did not incorporate this despite requesting this after review of their original manuscript.

**Response：**Thank you for the insightful and constructive feedback provided by the reviewers. In response, we have enriched our manuscript with pertinent supplementary data（AFC, Gravity and Parity）, which are distinctly highlighted in yellow for ease of reference within the revised article.

We appreciate for Editors/Reviewers’ warm work earnestly, and hope that the correction will meet with approval.

Once again， thank you very much for your comments and suggestions.

Sincerely,

Tianzhong Ma and Huizhen Li
